# Interactions between 14 Elements in the Human Placenta, Fetal Membrane and Umbilical Cord

**DOI:** 10.3390/ijerph16091615

**Published:** 2019-05-08

**Authors:** Karolina Kot, Danuta Kosik-Bogacka, Natalia Łanocha-Arendarczyk, Witold Malinowski, Sławomir Szymański, Maciej Mularczyk, Natalia Tomska, Iwona Rotter

**Affiliations:** 1 Department of Biology and Medical Parasitology, Pomeranian Medical University in Szczecin, Powstanców Wielkopolskich 72, 70-111 Szczecin, Poland; kotkarolina17@gmail.com (K.K.); nlanocha@pum.edu.pl (N.Ł.-A.); 2 Independent of Pharmaceutical Botany, Department of Biology and Medical Parasitology, Pomeranian Medical University in Szczecin, Powstańców Wielkopolskich 72, 70-111 Szczecin, Poland; 3 Department of Obstetrical and Gynecological Nursing, Pomeranian Medical University in Szczecin, Zolnierska 48, 71-210 Szczecin, Poland; witold05@op.pl (W.M.); sszymanski@o2.pl (S.S.); 4 Chair and Department of Human and Clinical Anatomy, Pomeranian Medical University in Szczecin, Powstanców Wielkopolskich 72, 70-111 Szczecin, Poland; maciej.mularczyk@pum.edu.pl; 5 Department of Medical Rehabilitation and Clinical Physiotherapy, Pomeranian Medical University in Szczecin, Zolnierska 48, 71-210 Szczecin, Poland; natalia.tomska@o2.pl (N.T.); iwrot@wp.pl (I.R.)

**Keywords:** trace elements, human, afterbirths

## Abstract

The aim of the study was to investigate relationships between the concentrations of macroelements (Ca), microelements (Cr, Cu, Fe, Mn, Mo, Ni, Sn, Sr, V, Zn) and heavy metals (Ag, Cd, Pb) in the placenta, fetal membrane and umbilical cord. ‪Furthermore, we examined relationships between the concentrations of these metals in the studied afterbirths and maternal age, gestational age, placenta parameters (breadth, length, weight) and newborn parameters (length, weight and Apgar score). This study confirms previously reported Zn-Cd, Pb-Cd and Ni-Pb interactions in the placenta. New types of interactions in the placenta, fetal membrane and umbilical cord were also noted. Analysis of the correlations between metal elements in the afterbirths (placenta, fetal membrane and umbilical cord) and biological parameters showed the following relationships: maternal age and Mn (in the fetal membrane); gestational age and Cr, Fe, Zn (in the fetal membrane), Ag and Cu (in the umbilical cord); newborn’s length and Sr (in the placenta), Ag (in the umbilical cord); newborn’s weight and Sr (in the placenta), Cu (in the fetal membrane), Ag (in the umbilical cord); Apgar score and Ca, Cr and Ni (in the umbilical cord); placenta’s length and Cr and Sn (in the fetal membrane), Cu (in the umbilical cord); placenta’s width and Mo, Pb (in the placenta) and placenta weight and Sr (in the placenta), Ag, Fe, Mn (in the fetal membrane). The results show the influence of metals on the placenta, mother and newborn parameters, and the same point indicates the essential trace elements during the course of pregnancy.

## 1. Introduction

During gestation, the fetus can be exposed to a number of toxic metals along with the nutrients and beneficial elements [1]. Due to the immaturity of the developing immune system, the rapid development of fetal organs and a higher absorption compared to adults, neonates are considered highly susceptible to chemical toxicants in the blood supply [2,3].

Placenta serves as an interface between maternal and fetal circulation, mediates nutrient transport and prevents toxic substances such as heavy metals from passing to the fetus. However, the human placenta does not block the passage of all toxic elements and some of them manage to pass through, posing a potential risk to human fetus [2,4,5]. Non-essential metals are able to cross biological membranes due to the fact that they possess similar molecular size and charge to that of essential metals, which is known as molecular mimicry [6,7]. It has been shown in animal studies that placental nutrient transport systems can also recognize xenobiotics as targets [8]. Therefore, the placenta has been identified as an indicator of fetal exposure to toxic metals [9]. Some studies have also investigated correlations between heavy metals concentration in the placenta and fetal growth and development, which may then lead to severe fetal damage [10,11].

Calcium (Ca) is an essential element for the formation of the skeleton. As a result of dystrophic changes in the placenta related to age, minor placental calcification is often observed in the late stages of gestation. This increased calcification of the placenta may have some negative consequences, as it may cause pathological maturation of the placenta and restrictions in fetus growth. During pregnancy, Ca is used to prevent preeclampsia by contributing to the normalization of blood pressure [12]. Molybdenum (Mo) is a trace element that is needed for important biological processes. Molybdenum compounds can cross the placental barriers. There are no reports showing the effects of Mo compounds on the mother or fetus [13]. Iron (Fe) is critical to fetal development because it is a necessary compound of hemoglobin and a cofactor of many enzymes regulating oxygen transport, cellular respiration, lipid metabolism and DNA synthesis [14]. Iron deficiency in the mother or placental dysfunction may increase the risk of preterm delivery, low birth weight, stillbirth and reduced iron stores in the newborn [15]. Zinc (Zn) and copper (Cu) are important elements that are needed for human growth and development [16]. Zinc is involved in metabolic and physiological processes that control cell growth, while Cu plays an important role in the absorption and metabolism of Fe [17]. Low levels of Zn and Cu are independently associated with a risk of low birth weight neonates. Decreased Cu levels were found in pregnant women with preeclampsia [18,19,20]. Manganese (Mn) is an essential antioxidant nutrient in pregnancy [21], but elevated Mn concentrations can also be associated with gestational hypertension [22], increased risks of neural tube defects [23] and reduced birth weight [24]. The function of strontium (Sr), a non-essential trace element, in the human body is not very well known. Only small amounts of Sr are transferred from the mother to the fetus through the placenta [25], but Sr has been implicated in the pathophysiology of preeclampsia [26]. Data on the effects of tin (Sn) on the fetus are very limited, but suggest that the possibility of a low level of transfer across the placenta [27]. Theuer et al. [28] observed no effects on the numbers of litters, resorptions, or live fetuses per litter in rats fed with a diet containing elevated levels of tin compounds, nor on mean placental and fetal weights. Chromium (Cr) is directly involved in the metabolism of carbohydrate, fats and protein. Low Cr intake has been associated with gestational diabetes mellitus. However, excess Cr exposure may cause congenital malformations, low birth weight and DNA damage in the fetus [29,30,31]. Nickel (Ni) has been associated with low birth weight [32], neural-tube defects [33], DNA damage in the fetus [34] and gestational age [35]. Lead (Pb) exposure can cause spontaneous abortions [36], congenital malformations [37], reduced birth weight [38] and length [39], gestational hypertension [40] and impaired neurodevelopment [41]. Cadmium (Cd) has been connected with decreased birth weight [42], premature delivery [43] and altered thyroid hormone status of newborns [44]. There have been no reports in humans about developmental effects induced by vanadium (V) or silver (Ag) in humans. However, some studies with rats and mice indicate that V can cross the placental barrier and accumulate in fetal membranes [45], while exposure to Ag nanoparticles results in the accumulation of nanoparticles in the placenta and fetal tissues, and induces an adverse pregnancy outcome [46].

This current study was performed to investigate (1) the concentration of macroelements (Ca), microelements (Cr, Cu, Fe, Mn, Mo, Ni, Sn, Sr, V, Zn) and heavy metals (Ag, Cd, Pb) in the human placenta, fetal membrane and umbilical cord, obtained from patients without complications during the pregnancy and delivery; (2) relationships between the analyzed elements in the placenta, fetal membrane and umbilical cord; and (3) the influence of the elements on biological parameters such as maternal age, gestational age, placental parameters (breadth, length, weight) and newborn parameters (length, weight and Apgar score).

## 2. Materials and Methods 

### 2.1. Ethics Statement

The use of the materials in the study was approved by the Bioethics Committee of the Pomeranian Medical University in Szczecin (KB-0012/76/14 on 13 October 2014). The patients participating in the study were informed of the course of the research project and they provided a written consent prior to participation. At every stage of the study, the patients had the possibility to withdraw participation.

### 2.2. Study Material

A total of 170 samples were obtained, comprising 81 placentas, 67 fetal membranes and 22 umbilical cords from 83 mothers aged from 17 to 44. The study involved women with a properly developing pregnancy and with the course of delivery without complications (i.e., preterm delivery, preeclampsia). More information about patients is presented in Table 1. The patient’s history and questionnaires provided information concerning age, gestational age, placental parameters (breadth, length, weight) and newborn parameters (length, weight, and Apgar score).

### 2.3. Preparation of Tissue Material for Analysis

The material was stored in a freezer at −27 °C until analysis. After complete defrosting to room temperature, samples of the placenta, fetal membranes and umbilical cords were dried to a constant mass at 105 °C. The dried samples were mineralized; more details of the analytical procedures are given by Kosik-Bogacka et al. [47,48]. Determination of Ag, Ca, Cd, Cr, Cu, Fe, Mn, Mo, Ni, Pb, Sn, Sr, V and Zn was performed by spectrophotometric atomic absorption in inductively coupled argon plasma (ICP AES), on a Perkin-Elmer Optima 2000 DV (USA). The concentrations of elements were expressed as mg/kg dry weight (dw) for Ag, Cd, Cr, Cu, Fe, Mn, Mo, Ni, Pb, Sn, Sr, V and Zn, and in g/kg dw for Ca.

### 2.4. Validation of Analytical Procedures

The accuracy of analytical labeling was controlled by determination of the elements in a certified material with a known concentration: NIST Standard Reference (SRM) 1577c Bovine Liver (National Institute of Standards and Technology). The mean concentrations of elements ranged from 94.6% to 106% of the reference values.

### 2.5. Statistical Analysis

Statistical analysis was carried out using Statistica PL software. Intergroup comparisons were performed using the Kruskal–Wallis test. The correlations were analyzed on the basis of Pearson’s correlation factor (*r*). Additionally, we used Tukey’s two-tailed test to detect outliners with a substantive approach. The significance level was *p* < 0.05.

## 3. Results

The average water content in the placenta, fetal membrane, and umbilical cord were approximately 83%, 85% and 98%, respectively.

The concentrations of analyzed elements in the placenta, fetal membrane and umbilical cord are presented in Table 2. Concentrations of elements in the placenta, fetal membrane and umbilical cord can be arranged in the respectively descending series:

Fe > Zn > Cu > Ca > Sr > Mn > Pb > Sn > Mo/Cr > Ni > Cd > V > Ag,

Fe > Zn > Cu > Ca > Sr > Mn > Pb > Sn > Mo > Cr > Ni > Cd > V > Ag,

Fe > Zn > Cu > Ca > Pb > Mn > Sn > Sr > Mo > Ni > Cr > V > Cd > Ag.

There were statistically significant differences in the concentrations of the elements between the studied materials. We observed significantly higher concentrations of Mo, Ni, Pb and V in the umbilical cord compared to the placenta and fetal membrane. The levels of Ag, Cd and Sn were similar in all studied materials.

Statistical analysis was performed in order to calculate possible correlations between concentrations of elements in the placenta, fetal membrane and umbilical cord. The results are presented in Table 3, Table 4 and Table 5.

Separate analysis for each of the afterbirth parts (i.e., placenta, fetal membrane, umbilical cord) was performed. The highest number of revealed correlations was observed in the fetal membrane, followed by placenta and umbilical cord. The strongest correlation (the highest coefficient) in the fetal membrane was found between Ni and Pb (0.90), whereas in the placenta and umbilical cord was between Ca and Sr (0.92) and Ni and V (0.94), respectively. Interestingly, in the umbilical cord and placenta, statistically significant relationships were only synergistic, whereas in the fetal membrane, both synergistic and antagonistic connections were significant (Table 6, Table 7 and Table 8).

Furthermore, in the study we determined correlations between the 14 elements in the human placenta, fetal membrane and umbilical cord, and the parameters of the mothers, newborns and placentas. The number of connections can be arranged in the following descending order: fetal membrane > umbilical cord > placenta. The results are presented in Table 9, Table 10 and Table 11.

## 4. Discussion

The distribution of the studied elements varies between the placenta, fetal membrane and umbilical cord. The placenta selectively allows transport of studied trace elements and heavy metals to the developing fetus. The placental barrier blocks the transport of toxic xenobiotic metals to the fetus.

The demand for macro- and microelements varies during pregnancy, and any imbalance between them can cause obstetric failure. According to the Polish Gynecological Society’s position on vitamin and trace element supplementation during pregnancy, a pregnant woman should supplement folic acid, Fe, Mg, Ca, I, omega-3 acids and vitamins A, E and D3 [49]. There is some data showing that supplementation, e.g., with vitamin D3, may reduce the risk of preeclampsia, gestational diabetes, low birthweight and preterm birth [50,51]. However, excess of some macro-, microelements and vitamins may play a detrimental role in fertility [52]. In the present research, we did not obtain information from all the studied patients about supplementation and that is why future research is needed to complement the findings of the research.

In our study, the levels of Pb, Ni, Mo, and V were significantly higher in the umbilical cord than in the placenta and fetal membrane. Contrary to our observations, Sakamato et al. [53] and Zhou et al. [54] found higher Pb concentrations in maternal blood compared to the umbilical cord blood, implying a possible passive transport of the elements from mother to fetus. The authors postulated limited placental protection from Pb [53,54]. Kutlu et al. [55] determined Pb concentrations in placental samples of smoking, passive smoking and nonsmoking mothers. The concentrations of Pb in the placenta obtained from the mothers in that study were similar to those noted in mothers who smoked 25 cigarettes per day (0.258) [55]. In our study, none of the patients admitted cigarette smoking, although it is estimated that the percentage of pregnant women exposed to passive tobacco smoking may be as high as 50% in some regions of Poland [56]. Exposure to tobacco smoke results in changes to macro- and microstructures of the placenta and the effectiveness of its functioning, which affect the quality of maternal fetus-placenta transport [57], and may explain higher Pb concentrations in the umbilical cord compared to the placental tissues.

There is not much information available on Ni concentrations in the placenta. Based on existing literature, the median concentration of Ni in the placenta appears to be 36 ng/g with a range of 9–62 ng/g dw, higher than in our study [9]. Klopov et al. [58] showed that Ni concentration in the umbilical cord blood was higher than in the maternal blood. We also found a higher concentration of Ni in the umbilical cord than in the placenta, thus confirming the ability of Ni to pass through the placental barrier.

The role of trace metals is to ensure the proper activity of biochemical and enzymatic reactions. Some trace elements and heavy metals have common chemical properties, so their metabolic interactions may increase the health hazard. We found a positive correlation between Cd and Zn in the placenta and fetal membrane. Similar results were obtained by Sabra et al. [59] and Mikelson et al. [60]. However, we did not notice such correlation in the umbilical cord, possibly due to the relatively small number of samples, thus the results warrant further confirmation in larger studies.

In our study, the strongest relationship was in the umbilical cord was between Ni-V, whereas in the placenta and fetal membrane the highest coefficient cofactors were noted between Ca-Sr and Ni-Pb, respectively. Mikelson et al. [60] also analyzed correlations between metals in the placenta tissues. The authors found the highest coefficient cofactor between Cu-Se followed by Se-Zn, Cu-Fe and Cu-Mn. In our study, we also found relationship between Cu-Mn in the fetal membrane and umbilical cord, and the coefficient cofactors were similar to those observed by Mikelson et al. [60]. However, we did not find correlation between Cu-Fe in any studied samples. Guo et al. [35] analyzed the Pb-Ni correlation in the placenta but the coefficient cofactor was lower than in our study. However, the authors analyzed placentas from a population living in an e-waste recycling town in China [35], therefore the comparisons should be interpreted with caution as the geographic location and environmental pollution are known to affect elemental exposure, uptake and distribution [61].

Premature birth is a complex and unresolved public health problem with a cause yet unknown. One of the crucial reasons for premature delivery is likely oxidative stress in trophoblastic placental tissues [62]. There are a number of studies concerning the effects of metals on oxidative and antioxidative processes. Strong positive correlations between Mn, Pb as well as Cd and malondialdehyde (MDA) concentration have been found [62,63], which suggests that these metals may be associated with increased formation of reactive oxygen species (ROS) and thus may produce oxidative stress in pregnant women leading to premature deliveries. The effects of ROS are counteracted by antioxidants such as glutathione (GSH), as mechanisms against the effects of oxidative stress are constituted by Cu/Zn dependent enzymes, which protect the placenta from damage. Increased levels of GSH have been found in full-term delivery samples compared to pre-term delivery [62]. In our study, we observed a positive relationship between gestational age and Zn level in the fetal membrane, and Cu concentration in the umbilical cord and gestational age. Moreover, we found positive correlations between Cr as well as Fe and gestational age in the fetal membrane and negative correlation between Ag levels in the umbilical cord and gestational age. Irwinda et al. [64] also found higher concentration of Fe, Cu and Zn in the placenta of term births than preterm births.

All changes concerning fetus development are reflected in morphological and functional disorders of the placenta. Recent studies have investigated the relationship between placental morphological changes and complicated pregnancies. Janthanaphan et al. [65] and Bortolus et al. [66] showed that placental weight increased with birth weight. In diabetic pregnancies, placental weight was higher than that in non-diabetic pregnancies [67]. The altered structure of placentas may also be compromised in the ability to transport xenobiotic element to the fetus. In our study, we determined the influence of trace metals on morphological parameters of the placenta. We observed correlations between Cu level in the umbilical cord, Cr and Sn level in the fetal membrane and placenta length. We also found relationships between Mo as well as Pb in the placenta and placenta width, and placenta Sr as well as Ag, Fe, Mn concentration in the fetal membrane and placenta weight.

Patients with complex heart defects and chromosomal abnormalities were observed to have lower fetal lengths [68]. Zheng et al. [69] also found that newborn length and weight was lower in an adverse pregnancy outcome group than that in the control group. The correlation analysis between biogenic and xenobiotic elements and anthropometric parameters showed negative relationships between Cu concentration in the fetal membrane and newborns weight. We observed a positive correlation between Sr level in the placenta and birth weight and length. We also noticed negative relationships between Ag concentration in the umbilical cord and newborn height and length. Mbofung and Subbarau [70] found positive correlation between placental Cu and birth weight, whereas Mikelson et al. [60] found negative correlation between Cu levels in the placental tissues and birth weight, but the relationship was not statistically significant. Mikelson et al. [60] also found negative relationships between the concentration of Mn and Pb in the placental tissues and birth weight, and the concentration of Cd as well as Mn and birth length. In our study, we did not confirm these correlations.

The Apgar score is a standardized index used to assess well-being at 1 and 5 minutes after birth, incorporates five elements: respiratory effort, heart rate, reflex irritability, muscle tone, and color [71]. Even though the Apgar score was originally not intended for the prediction of outcomes beyond the immediate postnatal period, at present multiple studies have examined the relation between the value of Apgar score and duration of low Apgar score and subsequent death or neurologic disability, due to the fact that a low Apgar score correlates with prenatal and perinatal adversities, and noted that a five-minute Apgar score <7 has a consistent association with prevalence of neurologic disability and with low cognitive function in early adulthood [72]. The correlation analysis between biogenic and xenobiotic elements and Apgar score showed a positive relationship between the concentration of Ca, Cr and Ni in the umbilical cord. It was found that Ca and Cr contributed to higher Apgar score. Moreover, our study suggested that in women with physiological pregnancy and without metal exposure, the placenta barrier blocks the transport of Ni to the fetus resulting in good health of the newborn.

The present study has several potential limitations. First, maternal diet and pre-pregnancy BMI as well as women’s BMI during pregnancy in order to monitor nutrients and food intake that influence metals concentration. Second, serum trace elements were not assessed using the study that could be beneficial to investigate the systemic changes of trace element status. Finally, it would be essential to examine the relationship between adverse maternal outcomes and the other micronutrients levels. Further analysis aimed at these points would be beneficial for assessment of the disturbance of trace element and mineral metabolism in women and their children.

## 5. Conclusions

Interactions between elements can affect the homeostasis of the mother and fetus, which may lead to unexpected biological effects. New types of interactions between metals in the placenta, fetal membrane and umbilical cord were noted. Although the results must be interpreted with caution, they show the influence of metals on the placenta, mother and newborn parameters, and the same point indicate the essential trace elements during the course of pregnancy, as well as improve children’s health.

The results of this study can be used to develop reference values for macro- and microelements in women with healthy pregnancies. Moreover, our study showed that measurement of Cu and Ag could be a promising biomarker for predicting the risk of low birth weight in clinical practice.

## Figures and Tables

**Table 1:** Information on maternal, newborn and placenta parameters included in the study (AM, arithmetic mean; SD, standard deviation; *n*, number).

Factors		
Maternal parameters		
Age	AM ± SD (range)	29 ± 5.4 (17–44)
Gestational age	AM ± SD (range)	39 ± 1.8 (27–41)
Number of pregnancies	primigravida	*n* = 28
	multigravida	*n* = 55
Cigarette smoking	yes	*n* = 0
	no	*n* = 83
Alcohol drinking	yes	*n* = 0
	no	*n* = 83
Working in heavy industry	yes	*n* = 0
	no	*n* = 83
Chronic disease (hypertension, gestational diabetes)	yes	*n* = 0
	no	*n* = 83
Drug addiction history	yes	*n* = 0
	no	*n* = 83
Cognitive impairment	yes	*n* = 0
	no	*n* = 83
In vitro fertilization	yes	*n* = 0
	no	*n* = 83
Place of residence	North-Western Poland	*n* = 30
	Central Poland	*n* = 53
Newborn parameters		
Gender	Female	*n* = 33
	Male	*n* = 50
Height (cm)	AM ± SD (range)	54 ± 3.2 (39–61)
Weight (g)	AM ± SD (range)	3494 ± 549.6 (1200–4850)
Apgar score	AM ± SD (range)	9 ± 0.9 (4–10)
Placenta parameters		
Length (cm)	AM ± SD (range)	18 ± 3.5 (10–29)
Breath (cm)	AM ± SD (range)	18 ± 3.6 (10–28)
Weight (g)	AM ± SD (range)	564 ± 172.7 (100–1000)

**Table 2:** Concentrations of Ag, Ca, Cd, Cr, Cu, Fe, Mn, Mo, Ni, Pb, Sn, Sr, V, and Zn in the human placenta, fetal membrane and umbilical cord (AM, arithmetic mean; K-W test, Kruskal–Wallis test; Ca concentrations are expressed in g/kg dw and Ag, Cd, Cr, Cu, Fe, Mn, Mo, Ni, Pb, Sn, Sr, V, as well as Zn levels are expressed in mg/kg dw).

Element	Placenta			Fetal Membrane			Umbilical Cord			K-W Test
	AM	Med.	Range	AM	Med.	Range	AM	Med.	Range	
Ag	0.006	0.003	0.001–0.080	0.005	0.003	0.001–0.050	0.007	0.003	0.003–0.049	*p* = 0.62
Ca	5.624	3.474	0.649–54.480	4.221	1.665	0.503–35.111	0.924	0.852	0.522–1.997	*p*<0.01
Cd	0.010	0.010	0.005–0.026	0.009	0.009	0.003–0.018	0.009	0.008	0.004–0.025	*p* = 0.08
Cr	0.064	0.060	0.034–0.174	0.070	0.057	0.034–0.785	0.036	0.032	0.020–0.069	*p* < 0.01
Cu	6.013	5.640	3.471–15.391	8.906	8.224	3.629–17.136	4.320	3.838	1.905–12.005	*p* < 0.01
Fe	640.726	619.710	192.127–1263.591	640.726	619.709	192.127–1263.590	567.285	496.154	115.080–2034.739	*p* < 0.01
Mn	0.446	0.442	0.230–1.210	0.772	0.650	0.332–2.630	0.349	0.306	0.158–0.638	*p* < 0.01
Mo	0.064	0.060	0.024–0.170	0.071	0.063	0.025–0.150	0.084	0.074	0.037–0.220	*p* < 0.01
Ni	0.028	0.023	0.013–0.098	0.030	0.025	0.013–0.070	0.040	0.036	0.018–0.123	*p* < 0.01
Pb	0.296	0.261	0.136–0.854	0.317	0.284	0.094–0.578	0.419	0.391	0.203–1.021	*p* < 0.01
Sn	0.281	0.285	0.145–0.400	0.271	0.261	0.173–0.450	0.291	0.285	0.208–0.418	*p* = 0.31
Sr	1.271	0.644	0.115–16.990	0.960	0.474	0.138–10.160	0.258	0.227	0.158–0.438	*p* < 0.01
V	0.008	0.007	0.005–0.017	0.008	0.007	0.006–0.015	0.010	0.010	0.007–0.014	*p* < 0.01
Zn	66.904	66.125	43.877–109.288	62.788	63.026	36.635–100.058	54.653	52.389	35.583–81.461	*p* < 0.01

**Table 3:** Pearson’s correlation coefficients between analyzed elements in the placenta and fetal membrane (*p* < 0.05).

	Ag	Ca	Cd	Cr	Cu	Fe	Mn	Mo	Ni	Pb	Sn	Sr	V	Zn
Placenta														
Ag	0.12	−0.06	−0.37	−0.27	−0.18	−0.09	−0.15	−0.23	−0.51	−0.39	0.12	−0.13	−0.01	−0.20
Ca	0.10	−0.03	0.35	0.01	0.55	−0.10	0.36	0.45	0.35	0.23	−0.26	0.00	0.18	0.07
Cd	−0.04	−0.19	0.34	0.16	0.39	−0.02	0.26	0.13	0.20	0.26	−0.24	−0.07	0.20	0.46
Cr	−0.15	0.11	0.31	0.44	0.40	−0.03	0.25	0.22	0.29	0.42	−0.35	0.12	0.58	0.49
Cu	0.32	−0.01	0.29	0.16	0.39	0.22	0.10	0.09	0.22	0.05	−0.01	−0.03	0.04	0.29
Fe	0.01	0.16	0.05	0.23	0.25	0.22	0.03	0.21	0.06	0.22	−0.23	0.22	0.10	0.21
Mn	−0.04	−0.01	−0.05	0.21	0.05	0.19	0.10	−0.23	−0.16	−0.18	0.19	0.02	−0.26	0.07
Mo	0.26	−0.12	0.07	0.32	0.15	0.05	0.05	0.09	0.04	0.12	0.32	0.12	−0.04	0.20
Ni	−0.01	−0.18	0.08	0.36	0.30	0.12	0.18	0.00	0.05	0.17	−0.07	0.06	−0.08	0.34
Pb	0.10	−0.14	0.01	0.26	0.23	0.08	0.15	0.01	0.02	0.11	0.11	0.03	−0.01	0.27
Sn	0.25	−0.01	−0.03	0.24	0.11	0.09	0.17	0.03	−0.04	0.15	0.47	0.11	−0.16	0.12
Sr	0.23	0.31	0.43	0.25	0.29	−0.08	0.29	0.38	0.30	0.37	−0.10	0.32	0.22	0.08
V	0.31	0.17	0.15	0.31	−0.04	0.13	−0.01	−0.11	−0.13	0.03	0.12	0.20	0.00	0.26
Zn	−0.15	−0.02	0.36	0.29	0.42	−0.01	0.27	0.27	0.43	0.32	−0.34	0.05	0.35	0.55
Fetal membrane														

**Table 4:** Pearson’s correlation coefficients between analyzed elements in the umbilical cord and fetal membrane (*p* < 0.05).

	Ag	Ca	Cd	Cr	Cu	Fe	Mn	Mo	Ni	Pb	Sn	Sr	V	Zn
Umbilical cord														
Ag	0.30	0.09	0.26	−0.60	−0.44	−0.98	−0.32	−0.43	−0.10	0.11	0.46	0.13	0.24	0.18
Ca	0.18	0.68	0.74	0.82	0.87	0.22	0.89	0.40	0.64	0.65	−0.24	0.50	0.50	0.60
Cd	0.26	0.18	0.55	0.62	0.37	0.06	0.07	0.51	0.62	0.55	−0.49	0.15	0.25	0.52
Cr	−0.42	0.28	0.51	0.68	0.71	0.24	0.69	0.52	0.50	0.50	−0.28	0.12	0.43	0.42
Cu	0.29	−0.24	0.01	0.22	−0.22	0.18	−0.09	0.32	0.32	0.17	−0.10	−0.60	0.18	0.38
Fe	−0.12	−0.10	0.29	0.47	0.50	0.06	0.41	0.05	0.16	0.16	−0.05	−0.10	0.07	0.79
Mn	−0.76	0.11	−0.06	0.48	0.45	0.40	0.63	0.09	0.14	−0.04	0.17	−0.34	0.06	0.52
Mo	0.18	−0.51	−0.65	−0.50	−0.57	−0.17	−0.59	−0.45	−0.53	−0.49	0.64	−0.60	−0.59	0.25
Ni	0.17	−0.36	−0.22	0.12	−0.17	−0.01	−0.18	0.05	0.08	−0.03	0.00	−0.64	−0.20	0.65
Pb	0.09	−0.45	−0.36	0.01	−0.34	−0.02	−0.40	−0.14	−0.05	−0.23	−0.02	−0.54	−0.38	0.39
Sn	0.05	−0.54	−0.48	−0.36	−0.68	−0.15	−0.67	−0.40	−0.39	−0.49	0.41	−0.48	−0.58	0.13
Sr	−0.12	0.12	0.19	0.25	0.50	−0.03	0.29	−0.25	−0.13	0.00	0.42	0.17	−0.20	0.75
V	0.06	−0.70	−0.94	−0.89	−1.00	0.21	−0.60	0.27	−0.99	−0.99	1.00	−0.44	−0.90	0.80
Zn	0.22	0.38	0.66	0.67	0.73	0.25	0.31	0.46	0.41	0.51	−0.16	0.23	0.19	0.81
Fetal membrane														

**Table 5:** Pearson’s correlation coefficients between analyzed elements in the umbilical cord and placenta (*p* < 0.05).

	Ag	Ca	Cd	Cr	Cu	Fe	Mn	Mo	Ni	Pb	Sn	Sr	V	Zn
Umbilical cord														
Ag	0.51	0.25	0.20	0.03	−0.41	0.08	−0.53	0.14	0.14	0.21	0.44	0.23	−0.06	−0.15
Ca	−0.69	−0.21	−0.71	−0.54	−0.22	−0.27	0.19	−0.56	−0.69	−0.65	0.72	−0.12	−0.64	−0.14
Cd	0.10	0.03	0.64	0.02	0.26	−0.24	0.26	0.67	0.54	0.64	−0.47	−0.02	0.56	0.25
Cr	−0.40	−0.16	−0.36	−0.01	0.07	0.07	0.18	0.09	−0.15	−0.16	0.33	−0.46	−0.26	0.73
Cu	0.37	−0.48	−0.01	−0.02	−0.38	−0.12	−0.10	−0.20	0.21	0.02	−0.02	−0.56	0.15	0.18
Fe	−0.22	0.02	−0.15	0.04	−0.04	0.49	−0.16	0.46	0.11	−0.07	0.16	−0.34	−0.12	0.36
Mn	−0.30	−0.04	−0.11	−0.15	−0.44	−0.24	−0.10	−0.09	0.14	0.07	0.52	−0.18	0.00	−0.12
Mo	0.29	0.29	0.48	−0.04	0.28	−0.36	0.21	0.14	0.30	0.43	−0.25	0.41	0.29	0.02
Ni	−0.80	0.21	−0.06	−0.33	0.34	−0.40	0.57	0.14	0.01	0.05	0.15	0.05	0.12	0.18
Pb	−0.19	−0.23	0.01	−0.58	−0.19	−0.59	0.55	−0.14	−0.03	0.09	0.11	−0.45	0.06	0.04
Sn	−0.30	0.06	−0.43	−0.07	−0.26	0.15	−0.24	−0.40	−0.59	−0.26	0.63	0.10	−0.64	−0.30
Sr	−0.52	−0.14	−0.68	−0.35	0.01	−0.15	0.06	−0.48	−0.63	−0.56	0.67	−0.07	−0.62	0.38
V	−0.20	−0.14	0.21	−0.40	0.08	−0.55	0.48	0.01	0.11	0.28	0.14	−0.19	0.12	0.23
Zn	0.26	0.47	0.49	0.56	0.58	0.03	0.20	0.72	0.57	0.57	−0.11	0.06	0.34	0.79
Placenta														

**Table 6:** Pearson’s correlation coefficients for metals found in the fetal membrane (*p* < 0.05).

	Ag	Ca	Cd	Cr	Cu	Fe	Mn	Mo	Ni	Pb	Sn	Sr	V	Zn
Ag	1.00													
Ca	−0.21	1.00												
Cd	−0.33	0.24	1.00											
Cr	−0.36	0.61	0.47	1.00										
Cu	−0.04	0.07	0.24	0.00	1.00									
Fe	−0.20	0.38	0.10	0.36	−0.05	1.00								
Mn	−0.01	−0.25	0.11	−0.09	0.18	−0.04	1.00							
Mo	−0.12	−0.23	0.40	−0.09	0.39	−0.14	0.32	1.00						
Ni	−0.29	−0.14	0.66	0.09	0.27	−0.10	0.27	0.72	1.00					
Pb	−0.21	−0.29	0.66	0.02	0.33	−0.16	0.32	0.79	0.90	1.00				
Sn	−0.03	−0.32	0.20	−0.29	0.38	−0.07	0.35	0.66	0.52	0.69	1.00			
Sr	−0.18	0.67	0.23	0.49	0.15	0.33	−0.14	0.10	0.01	−0.03	−0.15	1.00		
V	0.33	0.01	0.25	0.00	0.29	−0.08	0.16	0.36	0.23	0.47	0.36	0.30	1.00	
Zn	−0.22	0.43	0.43	0.69	0.15	0.41	−0.08	−0.16	0.07	−0.04	−0.24	0.45	0.11	1.00

**Table 7:** Pearson’s correlation coefficients for metals found in the placenta (*p* < 0.05).

	Ag	Ca	Cd	Cr	Cu	Fe	Mn	Mo	Ni	Pb	Sn	Sr	V	Zn
Ag	1.00													
Ca	−0.08	1.00												
Cd	−0.07	0.01	1.00											
Cr	−0.05	0.46	0.15	1.00										
Cu	−0.06	−0.23	0.30	0.19	1.00									
Fe	−0.09	0.13	0.09	0.28	0.16	1.00								
Mn	−0.24	0.09	0.29	0.22	0.55	0.14	1.00							
Mo	0.06	−0.12	0.54	0.08	0.32	−0.08	0.10	1.00						
Ni	0.02	−0.25	0.55	0.10	0.23	0.00	0.10	0.67	1.00					
Pb	−0.04	−0.02	0.45	0.33	0.23	0.04	0.16	0.62	0.67	1.00				
Sn	0.27	0.11	0.03	0.19	−0.10	0.11	0.07	0.20	−0.02	0.10	1.00			
Sr	−0.07	0.92	0.00	0.51	−0.15	0.06	0.01	−0.16	−0.14	0.09	0.04	1.00		
V	0.11	0.00	0.32	0.21	0.17	0.06	0.06	0.33	0.48	0.55	−0.01	0.04	1.00	
Zn	−0.11	0.00	0.35	0.63	0.39	0.17	0.29	0.29	0.26	0.33	−0.08	0.10	0.28	1.00

**Table 8:** Pearson’s correlation coefficients for metals found in the umbilical cord (*p* < 0.05).

	Ag	Ca	Cd	Cr	Cu	Fe	Mn	Mo	Ni	Pb	Sn	Sr	V	Zn
Ag	1.00													
Ca	−0.16	1.00												
Cd	0.45	0.38	1.00											
Cr	0.13	0.37	0.65	1.00										
Cu	−0.03	0.42	0.39	0.58	1.00									
Fe	−0.06	0.31	0.20	0.61	0.43	1.00								
Mn	−0.04	−0.03	0.03	0.07	0.54	−0.08	1.00							
Mo	0.07	0.51	0.53	0.38	0.29	0.47	0.03	1.00						
Ni	0.38	0.49	0.79	0.43	0.22	0.24	−0.01	0.77	1.00					
Pb	0.35	0.36	0.87	0.63	0.16	0.13	−0.01	0.65	0.84	1.00				
Sn	−0.32	0.19	−0.20	−0.23	−0.32	−0.06	−0.16	−0.15	−0.16	−0.01	1.00			
Sr	0.02	0.52	0.15	0.04	0.21	0.14	−0.18	0.01	0.12	0.01	−0.07	1.00		
V	0.28	0.39	0.77	0.29	0.19	0.05	0.11	0.72	0.94	0.87	−0.12	0.05	1.00	
Zn	0.20	0.22	0.28	0.18	0.42	0.12	0.35	0.19	0.36	0.12	−0.24	0.13	0.11	1.00

**Table 9:** Pearson’s coefficients for correlations between biological parameters of the mother, newborn, and placenta, and the concentrations of Ca, Cd, Cr, Cu, Fe, Mn, Mo, Ni, Pb V, and Zn in the placenta (*p* < 0.05).

Parameter	Ag	Ca	Cd	Cr	Cu	Fe	Mn	Mo	Ni	Pb	Sn	Sr	V	Zn
Maternal age	0.09	−0.08	−0.08	−0.11	−0.10	0.16	−0.14	0.05	−0.03	−0.01	−0.12	−0.15	−0.12	0.03
Gestational age	−0.11	0.11	0.17	0.07	0.02	−0.11	0.02	0.00	0.00	0.19	−0.07	0.11	0.14	0.08
Newborn height	−0.08	0.09	0.01	−0.06	−0.08	−0.12	0.04	−0.16	−0.10	−0.08	−0.1	0.24	−0.01	−0.04
Newborn weight	−0.10	0.07	−0.04	−0.12	−0.15	−0.18	−0.07	−0.08	−0.01	0.03	−0.01	0.24	0.16	−0.18
Apgar score	0.05	−0.10	0.01	−0.04	0.04	−0.02	0.03	0.11	0.13	−0.01	0.03	−0.21	0.03	−0.1
Placenta length	−0.05	0.01	−0.15	−0.04	−0.16	0.00	−0.19	−0.05	−0.01	−0.08	−0.17	0.18	0.16	−0.06
Placenta width	0.18	0.02	−0.20	−0.16	−0.17	−0.15	−0.08	−0.24	−0.17	−0.26	−0.1	0.03	0.14	−0.17
Placenta weight	−0.01	0.13	0.05	0.07	−0.01	−0.14	−0.07	0.05	0.11	−0.14	−0.18	0.25	0.24	0.13

**Table 10:** Pearson’s coefficients for correlations between biological parameters of the mother, newborn, and placenta, and the concentrations of Ca, Cd, Cr, Cu, Fe, Mn, Mo, Ni, Pb V, and Zn in the fetal membrane (*p* < 0.05).

Parameter	Ag	Ca	Cd	Cr	Cu	Fe	Mn	Mo	Ni	Pb	Sn	Sr	V	Zn
Maternal age	−0.13	0.04	−0.15	−0.16	0.06	0.09	−0.25	−0.12	−0.07	−0.20	−0.15	−0.11	−0.18	−0.14
Gestational age	−0.03	0.02	0.22	0.36	−0.12	0.27	0.13	0.00	0.04	0.05	−0.10	0.18	0.11	0.25
Newborn height	−0.14	−0.14	0.09	0.09	−0.12	0.12	0.18	0.08	0.17	0.13	0.14	0.03	0.12	0.16
Newborn weight	−0.18	−0.25	0.09	0.08	−0.36	0.06	−0.05	0.07	0.04	0.14	0.04	−0.10	−0.14	−0.02
Apgar score	0.26	−0.05	−0.12	−0.09	−0.08	0.07	0.10	−0.08	−0.09	−0.07	0.05	0.20	0.14	−0.06
Placenta length	−0.07	0.26	−0.01	0.25	−0.19	0.20	−0.22	−0.22	−0.12	−0.20	−0.35	0.25	−0.28	0.09
Placenta width	0.05	−0.08	0.05	−0.06	0.11	−0.19	−0.24	0.05	0.01	0.08	−0.01	0.09	0.09	−0.07
Placenta weight	−0.28	0.12	0.02	0.23	−0.06	0.29	−0.35	−0.13	−0.10	−0.12	−0.18	0.18	0.03	0.23

**Table 11:** Pearson’s coefficients for correlations between biological parameters of the mother, newborn, and placenta, and the concentrations of Ca, Cd, Cr, Cu, Fe, Mn, Mo, Ni, Pb V, and Zn in the umbilical cord (*p* < 0.05).

Parameter	Ag	Ca	Cd	Cr	Cu	Fe	Mn	Mo	Ni	Pb	Sn	Sr	V	Zn
Maternal age	0.24	−0.17	−0.22	−0.38	−0.23	−0.27	0.00	0.00	−0.01	0.00	0.05	−0.14	0.17	−0.19
Gestational age	−0.53	0.16	−0.06	0.29	0.57	0.36	0.40	−0.08	−0.16	−0.18	0.20	0.06	−0.17	0.15
Newborn height	−0.53	0.12	0.03	0.29	−0.08	0.26	−0.31	−0.12	−0.16	0.02	0.24	0.08	−0.27	−0.10
Newborn weight	−0.55	−0.09	−0.23	−0.20	−0.16	−0.13	−0.07	−0.34	−0.23	−0.17	0.31	0.06	−0.24	−0.03
Apgar score	−0.21	0.53	0.54	0.61	0.37	0.12	0.09	0.24	0.50	0.44	−0.07	0.13	0.36	0.40
Placenta length	−0.35	0.13	0.07	0.33	0.53	0.20	0.29	0.08	0.07	0.17	−0.06	0.04	0.09	0.13
Placenta width	−0.27	0.00	−0.13	0.05	0.10	0.09	−0.25	−0.04	−0.09	−0.06	0.13	−0.09	−0.23	−0.02
Placenta weight	−0.10	−0.06	0.11	0.33	0.35	0.16	−0.17	−0.23	−0.11	0.00	−0.13	0.10	−0.15	0.02
